# Oscillatory cAMP cell-cell signalling persists during multicellular *Dictyostelium* development

**DOI:** 10.1038/s42003-019-0371-0

**Published:** 2019-04-23

**Authors:** Gail Singer, Tsuyoshi Araki, Cornelis J. Weijer

**Affiliations:** 1 Division of Cell and Developmental Biology, School of Life Sciences University of Dundee, Dundee, DD1 5EH UK; 2 Present Address: Department of Materials and Life Sciences, Sophia University, 7-1 Kioi-cho, Chiyoda-ku, Tokyo, 102-8554 Japan

**Keywords:** Biological techniques, Cell biology, Developmental biology

## Abstract

Propagating waves of cAMP, periodically initiated in the aggregation centre, are known to guide the chemotactic aggregation of hundreds of thousands of starving individual *Dictyostelium discoideum* cells into multicellular aggregates. Propagating optical density waves, reflecting cell periodic movement, have previously been shown to exist in streaming aggregates, mounds and migrating slugs. Using a highly sensitive cAMP-FRET reporter, we have now been able to measure periodically propagating cAMP waves directly in these multicellular structures. In slugs cAMP waves are periodically initiated in the tip and propagate backward through the prespore zone. Altered cAMP signalling dynamics in mutants with developmental defects strongly support a key functional role for cAMP waves in multicellular Dictyostelium morphogenesis. These findings thus show that propagating cAMP not only control the initial aggregation process but continue to be the long range cell-cell communication mechanism guiding cell movement during multicellular *Dictyostelium* morphogenesis at the mound and slugs stages.

## Abstract (web summary)

Gail Singer et al. demonstrate that cAMP waves are periodically initiated in the tip and propagate backward during multicellular *Dictyostelium* morphogenesis. This study suggests that propagating cAMP serves as the long-range intercellular communication signal beyond the initial aggregation process.

## Introduction

Long range cell-cell communication is thought to coordinate critical cell movements during the development of multicellular organisms. A well studied example of long range chemical cell-cell communication is the cAMP relay system controlling the chemotactic aggregation of hundreds of thousands of starving *Dictyostelium* *discoideum* cells into multicellular aggregates^1^. *Dictyostelium* cells live as single amoebae in the leaf litter of the soil where they feed on bacteria. Under starvation conditions up to a million single cells enter a multicellular developmental phase. Starving cells aggregate into multicellular aggregates that transform via mound and migrating slug stages into fruiting bodies, consisting of a stalk supporting a head of spores.

The aggregation of starving *Dictyostelium* *discoideum* cells occurs via chemotaxis guided by propagating waves of the chemoattractant cAMP. During early aggregation, cells in aggregation centres periodically release cAMP which is detected and relayed outward by surrounding cells. Cells move up the cAMP gradients during the rising phase of the waves resulting in their periodic movement towards the aggregation centre^2^. Variations in initial cell density, amplified by the increase in cell density during the first few waves of aggregation, lead to the formation of bifurcating aggregation streams, a phenomenon known as a streaming instability^3^. cAMP waves now primarily propagate through these streams from the aggregation centre outward, directing the collective cell movement of highly polarised cells, towards the aggregation centre resulting in the formation of the mound. During aggregation the cells start to differentiate into prestalk and prespore cells, precursors of the stalk cells and spores of the fruiting body. In the mound the prestalk cells sort out from the prespore cells guided by chemotactic signals to the top of the mound to form the tipped mound^4,5^. The tipped mound transforms into a migratory slug with prestalk cells in the front and prespore cells in the back. Under conditions of high light and low humidity the slug transforms into a fruiting body^1^.

The mechanisms of cAMP relay and chemotactic cell movement during early aggregation have been widely studied and the underlying molecular mechanisms are understood in considerable detail^6,7^. As a result of starvation induced changes in gene expression, cells start to express critical components of the cAMP detection, amplification and breakdown machinery that underlie the cAMP oscillations. Extracellular cAMP is detected via G protein coupled cAMP receptors, upon stimulation of the receptors this results in a signal transduction chain that leads to the activation of two processes, activation of a specific transmembrane adenylyl cyclase (AcA) that produces cAMP and a slower adaptation process that results in inhibition of cyclase activation^8^. The intracellular cAMP is secreted to the outside, where it stimulates the cAMP receptors sustaining the cAMP amplification, until the adaption process shuts this amplification cycle down^9,10^. cAMP is continuously degraded by a secreted cAMP phosphodiesterase resulting in a decay of extracellular cAMP, once production stops. This reduction in extracellular cAMP allows the cells to resensitise^11^. These processes result in oscillatory cAMP production in well stirred cell suspensions or to intricate spatio temporal propagating cAMP waves patterns when the cells are distributed on a substrate^12,13^. These propagating cAMP waves that control the aggregation process were first detected as light scattering waves caused by the periodic locally synchronised cell movements during the rising phases of the cAMP waves^14^. The period of the waves decreases during aggregation from initially 8 min to 2–3 min in the late stages of aggregation^15^. Analysis of these light scattering waves have been used extensively to characterise mutants in cell-cell signalling and chemotaxis^16^. Snapshots of cAMP waves have been measured via cAMP isotope dilution fluorography in fixed preparations of cells^13^. More recently it has become possible to detect these cAMP waves in living cell populations using cAMP specific fluorescence resonance energy transfer (FRET) reporter constructs based either upon the cAMP dependent protein kinase or the mammalian Rap1GEF, Epac (exchange protein directly activated by cAMP) during the early stages of aggregation^17–19^.

Propagating light-scattering waves associated with periodic movement of cells have been shown to persist from the early aggregation stages in aggregation streams to mounds and slugs, suggesting the presence of propagating chemotactic signals^20,21^. Furthermore localised cAMP injections have been shown to interact with these optical density waves in mound and slug stages of development further suggesting that these optical density signals in late aggregates and mounds are cAMP waves^21,22^. The aggregation stage adenylyl cyclase that controls aggregation continues to be expressed in the mound, slug and culmination stages, mainly in the prestalk cell population^23^. Experiments with a temperature sensitive acaA construct have shown that optical density waves in aggregates and mounds as well as slug migration are dependent on active AcA^24^. This suggests that this adenylyl cyclase continues to produce cAMP signals in mound and slug stages guiding chemotactic movement of cells in these stages of development. On the other hand it has been argued that cAMP oscillations are not required for slug migration, since it was reported that an acaA knockout mutant over expressing the cAMP dependent protein kinase catalytic subunit can form slugs and fruiting bodies^25^.

However so far no direct measurement of cAMP waves in these multicellular stages of development have been reported. Therefore it remains an open question whether the optical density waves observed during the post aggregation stages of development reflect cAMP waves as they do during early aggregation. To address this question we have adopted a highly sensitivity Epac1 based cAMP-FRET sensor for use of measurement of intracellular cAMP dynamics in *Dictyostelium*^26^. We have now visualised cAMP waves in aggregation streams, mounds and slugs and measure their dynamics during development. Measurement of aberrant cAMP signalling dynamics in three mutants, the aggregation stage adenylyl cyclase AcA, the internal phosphodiesterase RegA and the cell contact molecules TgrB1 and TgrC1, all with defects in multicellular development, strongly support a functional role for cAMP propagation in multicellular *Dictyostelium* development.

## Results

### Characterisation of a cAMP FRET sensor in *Dictyostelium*

To measure in vivo cAMP wave dynamics during multicellular *Dictyostelium* development we adapted a mammalian Epac1-based FRET construct H74 for expression in *Dictyostelium*^27^. This construct had been optimised for FRET measurements based on changes in fluorescence life-time upon cAMP binding in mammalian cells by using Turquoise2 as the donor and a tandem split Venus acceptor. We modified this construct for use in *Dictyostelium* by replacing the turquoise2 with *Dictyostelium* codon optimised version of ECFP or Turquoise2. Additionally we introduced a high affinity mutation Q270E^27,28^ in the constructs. The constructs were expressed under the control of the actin 15 promoter as described in methods. Initial experiments showed that life time measurements, with a time resolution good enough to detect the dynamics of the cAMP waves, had too low signal to noise ratios to provide reliable measurements in larger tissue structures such as mounds and slugs. Therefore we resolved to performing measurements using the more widely used ratio-metric FRET technique on either a confocal microscope or a fluorescence widefield microscope. The high affinity construct had an EC_50_ of ~0.2 uM for cAMPS in *Dictyostelium* cell lysates expressing the EPAC construct shown in (Supplementary Figure 1).

We observed that the cellular localisation of the Epac1 based cAMP FRET probe is not distributed homogeneously in the cell. A sizeable amount of the probe is found in the cytoplasm, but we also detect many bright fluorescent particles in the cell (Supplementary Figure 2, Supplementary Movie 1). However these particles move so fast that they are not in the same location for the 100 msec that it takes to acquire two successive frames at the blue and green emission wavelengths. Therefore we are not able to say unambiguously whether individual particles contribute measurably to the observed FRET oscillations. However filtering the raw image data to retain only the high intensity values associated with the particles still produces oscillations that are reflective of the general oscillations measured over the whole cell. Therefore it appears likely that these high intensity particles report changes in cAMP. At the moment it is not clear whether these highly fluorescent spots are associated with specific structures in the cell and if so what these structures are. They could even be vesicles that have been proposed to be involved in the production and secretion of cAMP^29^, although we do not find a clear localisation of these vesicles in the back of the cell, as has been reported for vesicles involved in cAMP secretion. For the moment we are satisfied that the FRET signal integrated over a cell gives a good representation of its cAMP dynamics.

### cAMP oscillations in the streaming aggregates

**Fig. 1:**
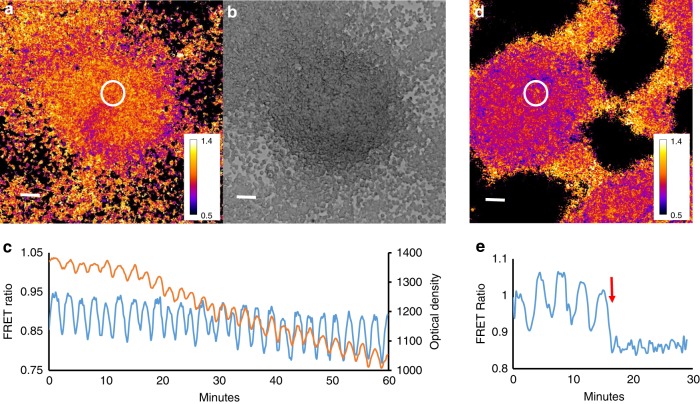
cAMP and optical density waves during late aggregation. **a** cAMP FRET ratio of a late aggregate. **b** Optical density image of the same aggregate as shown in (**a**). **c** FRET ratio and optical density changes as function of time measured in the circle region shown in **a**. Correlation analysis has shown that the FRET trace lags ~30 s to the optical density trace. Note the asymmetry of the FRET signal, the curves peak down which is also seen as a wide region of high FRET ratios and small band of lower FRET values (purple). **d** FRET image of mound on a black millipore filter. **e** Time trace of cAMP oscillations before and after exposure to 5 mM caffeine measured in the white circled area shown in **d**. FRET oscillations stop immediately after caffeine exposure at t = 15 min and there is an immediate decrease in the FRET ratio. White scale bar in images is 50 µm. The results are representative for over 30 experiments with wild-type cells and 10 experiments with caffeine performed on different days

To validate the cAMP probe for in vivo measurement of cAMP dynamics in larger multicellular structures we performed measurements of cAMP dynamics during the transition from the streaming aggregate to early mound stages of development. We reliably detected cAMP waves at these stages (Fig. 1a, b, Supplementary Movie 2). Simultaneous measurement of both light scattering and cAMP signals showed that there is a small phase lag between the cAMP waves and light scattering signal (Fig. 1c). Cross correlation calculations show that the cAMP waves in Fig. 1b are lagging the light scattering signal by 30 s. The lag is variable depending on the position of measurement and the stage of aggregation and typically varies between 30 and 150 s in these experiments.

**Fig. 2:**
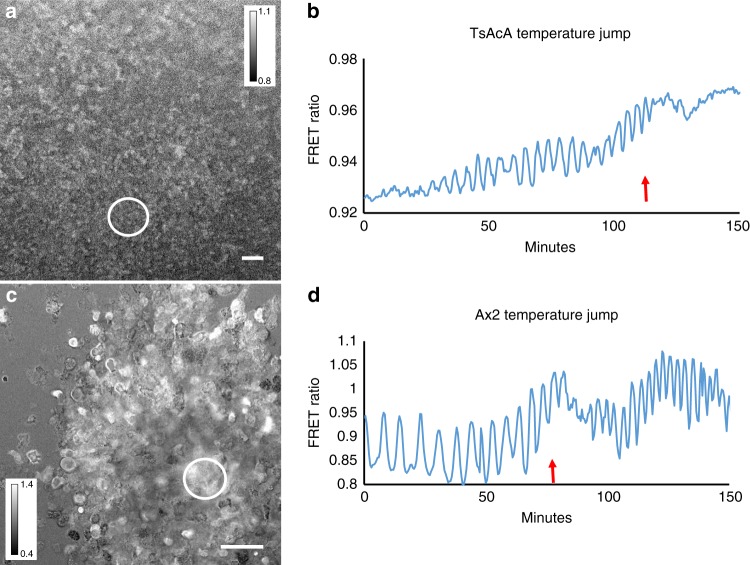
Effect of temperature shift of FRET oscillations in a temperature sensitive AcA mutant and in the parent strain Ax2. **a** FRET image of early aggregate of tsACA mutant expressing the EPAC construct. **b** Time trace measured in tsACA mutant at the circle position shown in **a** upon shift of the permissive temperature of 21 to 28 ℃ at the position of the red arrow. Note the rapid disappearance of the oscillations. The results are representative for 8 experiments performed with the tsACA mutant on different days. **c** FRET image of control Ax2 cells expressing the EPAC FRET construct. **d** Time trace measured in the Ax2 parent at the circle position shown in **G** upon shift of the permissive temperature of 21 to 28 ℃ at the position of the red arrow. The signalling period decreases with increasing temperature from 5 to 2.5 min. White scale bar in images is 50 µm. The results are representative for 14 experiments performed on different days

The cAMP waves observed at the early mound stage are inhibited almost instantaneously by application of 5 mM caffeine (Fig. 1d, e, Supplementary Movie 3), which is in agreement with biochemical data on early aggregation stage cells and measurement of the effects of caffeine in the optical density waves^15,30^. This makes it highly likely that the cAMP waves in the mounds are produced by the aggregation stage adenylcyclase, since caffeine has been show to inhibit the activation of this cyclase at the early stages of development^31^. To test the involvement of this cyclase directly we performed measurements of cAMP oscillations in a temperature sensitive adenylcyclase mutant tsAcA^24^. We found that we could only express low levels of the Epac1 based cAMP reporter in this strain. Selection of higher expression levels resulted in strains that failed to aggregate. This likely indicates that the tsAcA mutant produces less cAMP than wild-type at the permissive temperature and that expression of a cAMP chelating agent such as the Epac1 based cAMP FRET sensor blocks differentiation. However low levels of expression were tolerated and the cells were able to aggregate and form mounds, but not slugs at the permissive temperature of 21 °C, while still being able to report cAMP levels. Shifting aggregating *tsacaA* cells to the restrictive temperature of 28 °C during aggregation resulted in a rapid inhibition of the FRET oscillations, while the Ax2 parent strain continued to show cAMP oscillations at increased frequency at the restrictive temperature (Fig. 2, Supplementary Movie 4, Supplementary Movie 5). These experiments clearly show the key role of AcA in the generation of cAMP oscillations at late aggregate and mound stages and reinforce the key role for AcA in slug formation and slug migration.

**Fig. 3:**
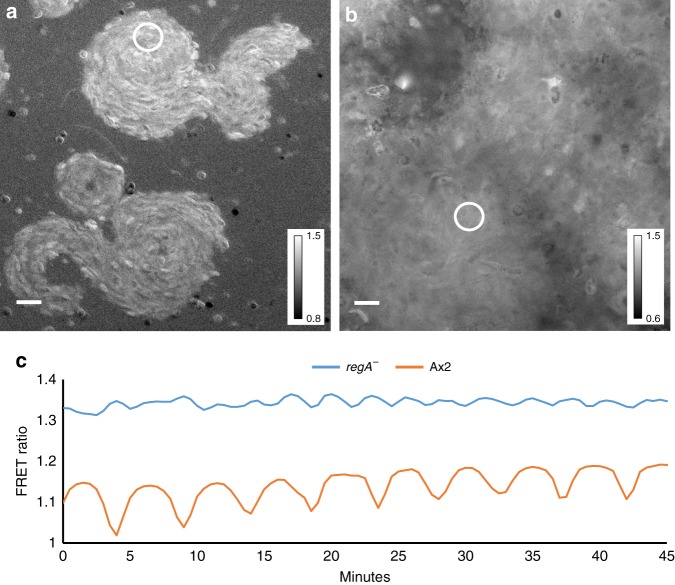
cAMP signalling in r*egA*^*−*^ and its parent strain Ax2 **a** FRET image of 8 h streaming aggregate of *regA*^*-*^ mutant. **b** FRET image of 8 h streaming aggregate of Ax2. **c** Time traces of FRET oscillations measured in the circled regions in the *regA*^−^ mutant (blue curve) and in Ax2 (red curve). Note the high FRET ratio and small amplitude and shorter period of the oscillations in *regA*^*−*^ compared to Ax2. These experiments are representative for results obtained in four independent experiments with *regA*^*−*^ performed on different days. White scale bar in images is 25 µm

Another key component known to be involved in the control of intracellular cAMP is the two component phosphodiesterase RegA^32^. RegA is strongly upregulated at the late aggregate mound stage and the *regA*^−^ mutant produces small aggregates and mounds, a few of which transform into small fruiting bodies with thick stalks. We found that expression of the cAMP FRET construct in this strain resulted in measurable but very low amplitude FRET oscillations on a high basal FRET ratio during aggregation and in the mound stages of development (Fig. 3a, c, Supplementary Movie 6). Although absolute calibration of the FRET probes in vivo is difficult, the higher FRET ratios observed compared to wild-type cells of similar stages of development (Fig. 3b, c) show higher basal levels of cAMP in the *regA*^−^ mutant indicating that RegA is a key component involved in the control of intracellular cAMP levels. It is interesting to note that the period of the oscillations at these stages are faster (~2.5 min) than observed in wild-type strains (~4 min) at these stages of development. This is in agreement with the observation of increased frequency of optical density waves in this mutant^33^. Thus altogether these measurements show that cAMP oscillations can be measured using the Epac1 based FRET probe and that their dynamics in wildtype and mutant strains is accurately reflecting the previously studied optical density waves in these strains.

### Correlating cAMP signalling and cell movement

**Fig. 4:**
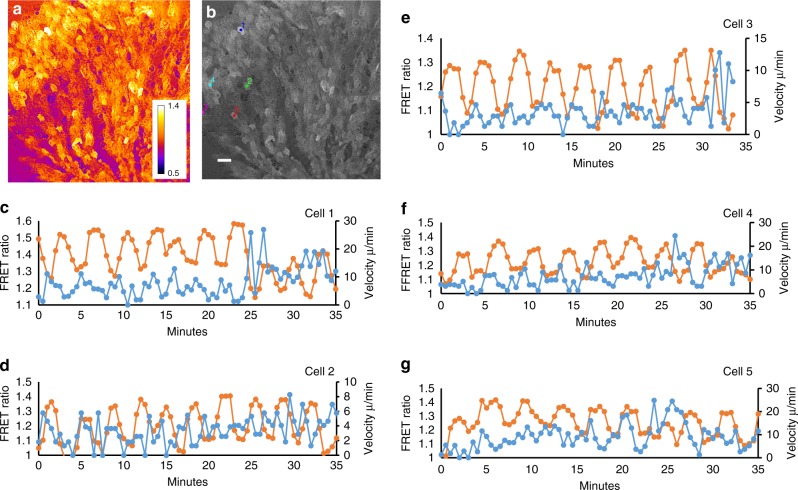
Correlation between cAMP signalling and movement of individual cells in an aggregation stream. **a** FRET ratio image of end of aggregation stream. **b** Image of stream with starting position of five cells that have been followed for around 35 min. **c**, **d**, **e**, **f**, **g** cAMP measurement (orange) and instantaneous rate of movement (blue) of cells 1, 2, 3, 4, 5, respectively. Lags calculated from the cross correlations between the FRET and velocity signals over the length of the tracks are −2.5, −1.5, −2.5, −2.5 and −3 min respectively for cells 1–5. This indicates that the cells maximal velocity is reached ~2.5 min before the internal cAMP concentration reaches its peak. White scale bar 50 µm. The experiments are representative for three experiments performed on different days

Detailed analysis of cellular behavioural responses in optical waves during the early aggregation have shown that cells orient and polarise during the initial rising part of the wave, stay polarised and keep moving during main part of the rising phase, round up and stop moving at the top of the wave followed by a reappearance of lateral pseudopods and random movement during the falling phase of the wave. Furthermore the *regA*^−^ mutant has been shown to be defective in suppression of lateral pseudopods and show defective myosin localisation^34^. This has led to the suggestion that internal cAMP might be involved in the control of the chemotactic movement cycle^35^. It is however unknown how the internal cAMP levels correlate exactly with the movement cycle especially when the cells are in close contact. To get further insight into this we have correlated the movement of individual cells with their internal cAMP levels, by tracking individual cells and measuring their cAMP levels (Fig. 4, Supplementary Movie 7). The results show that the oscillations in an individual cell’s cAMP is typically more regular than its instantaneous rate of movement. We have illustrated this for five randomly chosen cells in an aggregation stream. Cross correlation analysis between the cAMP oscillations and the cell’s velocity shows that the velocity peaks ~2.5 min before the internal cAMP reaches its maximum, implying that cells start to move as soon as they detect a gradient of cAMP. There is however considerable variation in the correlation between changes in movement speed and changes in internal cAMP between different cells in the same aggregation stream and even within a single cell at different times during aggregation. In some cases cells move fast when the cAMP levels are low and slow down when the internal cAMP levels are high, however sometime later the cell may move in synchrony with its own cAMP cycle, i.e. move fast when cAMP is high and slow when cAMP is low. These data are representative for observations over several independent experiments. The rate of movement is likely governed by a complex combination of the local external cAMP gradient, interactions with neighbouring cells and internal cAMP.

### Complex cAMP oscillatory behaviour in mounds

During the transition of the streaming aggregate to the mound stage we frequently observe rather complex changes in signalling dynamics (Supplementary Movie 8). Since it is known that during differentiation prespore cells down regulate expression of AcA it could be that this change in frequency reflects the differentiation of the cells in precursors of the stalk and spore cells and could even be related to the sorting of these cell types that occurs at these stages. To test this directly we made an expression construct where the Epac cAMP sensor was expressed under the control of the prestalk specific *pstA* promoter^36^. The expression of the construct appeared too late in development to actually observe the sorting process in sufficient detail to correlate changes in signalling with sorting directly, but we have been able to use this construct to measure oscillations in slug tips (see below).

**Fig. 5:**
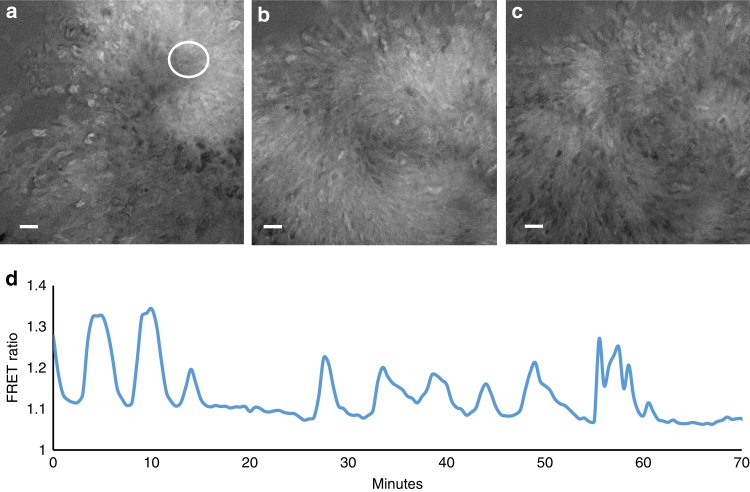
Complex cAMP signalling dynamics in aggregating *tgrB1*^*−*^*/tgrC1*^*−*^ cells. **a**, **b**, **c** Three successive images taken 15 min apart showing changes in signalling geometry in a *tgrB1*^*−*^*/tgrC1*^*−*^ mutant. **a** FRET ratio image showing single spiral wave. **b** Same aggregate as in **a** showing a two armed spiral. **c** Same aggregate as **a**, **b** showing a 4–5 armed spiral. **d** Time trace of evolution of FRET signal measured in the white circled area in **a**. White scale bar 50 µm. The results are representative for 10 experiments performed with the *tgrB1*^*−*^*/tgrC1*^*−*^ double null mutant on different days

**Fig. 6:**
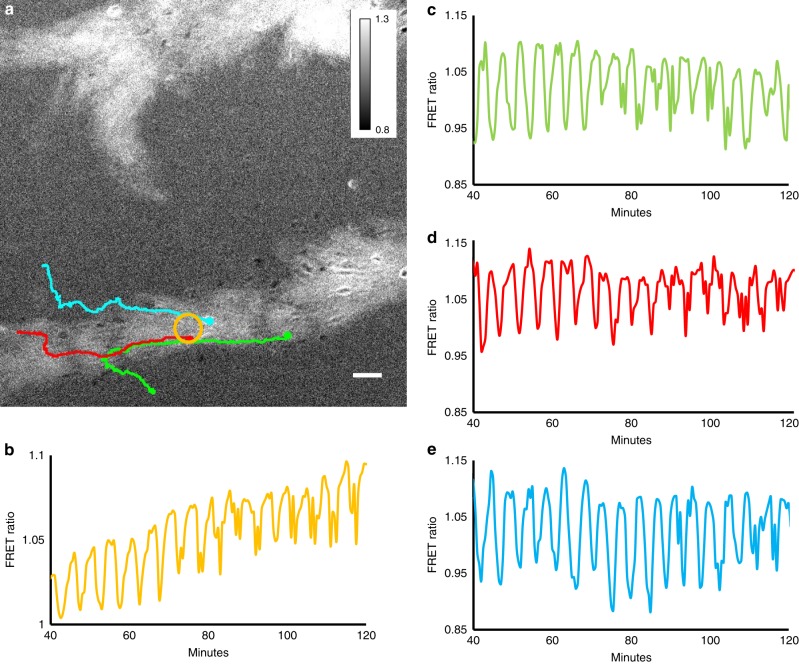
*tgrB1*^−^*/tgrC1*^*−*^ aggregation stream showing development of complex signalling dynamics. **a** FRET signal showing pairs of waves in aggregation stream and movement tracks of three cells whose cAMP signal was measured. **b** FRET image as function of time measured in the area by the yellow circle in **a**, **c**, **d**, **e** cAMP oscillations measured in the green, red and blue cells respectively As function of time. Note the change from regular slower oscillations to fast oscillations from around 70 min in the experiment onwards. White scale bar 50 µm. The results are representative for six experiments performed with the *tgrB1*^−^*/tgrC1*^−^ double null mutant strain performed on different days

A class of mutants that are suspected to have changes in cAMP signalling at the aggregate to mound transition are mutants in the Tgr contact molecules that appear to act as a ligand receptor pair and are involved in self recognition and kin selection^37,38^. These molecules are required for development and may mediate adhesion and/or cell-cell signalling as part of a kin selection system. *tgrB1*^−^*/tgrC1*^−^ mutants are arrested at the mound stage of development^39^. Mixing of incompatible Tgr allotypes results in sorting of these strains in mounds and slugs. Cells lacking the founding member of this family *tgrC1* (LagC) have been described to undergo cycles of aggregation, dissociation and re-aggregation^40^. These observations could reflect problems in cAMP cell-cell signalling. We find that *tgrB1*^*−*^*/tgrC1*^*−*^ mutants show very complex signalling dynamics during aggregation. Signalling modes can switch suddenly between slow and fast signalling almost instantaneously (Fig. 5a–d, Supplementary Movie 9). Furthermore in aggregation streams we observed complex modes of signalling where slower oscillations would be interspersed with high frequency signalling, giving rise to the formation of wave packets moving down aggregation streams (Fig. 6, Supplementary Movie 10). In some of these cases the bands can travel as pairs or groups of three separated by longer periods of inactivity. The interval between successive low FRET phases can be as short as one minute. We had noted this type of complex behaviour in the past in some streamer F mutants where optical density waves were seen to be moving in groups^41^. Since at that time it was not possible to measure cAMP signalling directly at the individual cell level we could not discriminate whether these signals were due to complex gating behaviour in populations of coupled oscillators or complex transitions in oscillatory behaviour in individual cells. However measurements of complex cAMP oscillations in individual cells in the *tgrB1*^−^*/tgrC1*^−^ mutant now clearly demonstrates that individual cells can indeed readily switch between low and high frequency oscillations, indicating that the underlying cAMP oscillator can show very complex dynamics.

### cAMP waves in slugs

**Fig. 7:**
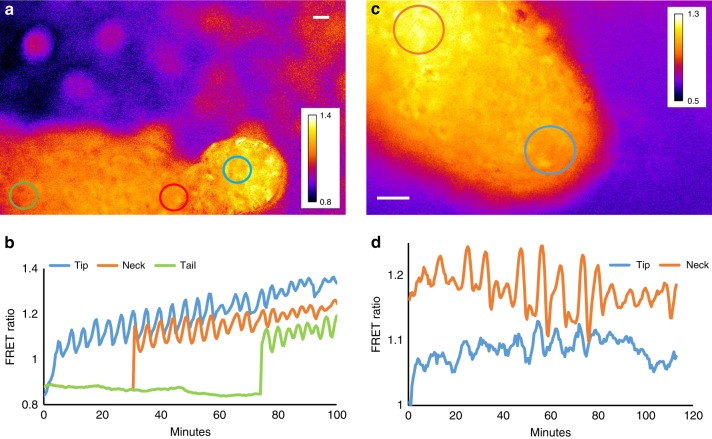
FRET ratio measurement of cAMP oscillations in different regions of young slugs. (**a**) FRET ratiometric image of confined slug migrating from left to right. **b** FRET ratio measured in the slug tip, neck and tail as a function of time. Measurements were performed by averaging areas in the blue, red and green areas respectively which tracked the movement of the tip neck and tail region as indicated in **a**. **c** FRET ratio of slug tip at higher magnification. **d** FRET traces measured in tip and neck regions of this slug over time as indicated by the blue and red circles in **c**. White scale bar 50 µm. The experiments are representative for over 15 experiments performed with slugs migrating under confined conditions

**Fig. 8:**
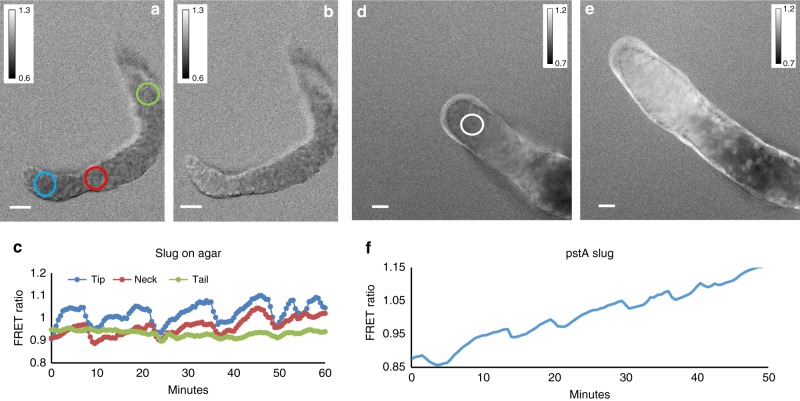
FRET ratio measurements of cAMP oscillations in slugs migrating on top of agar in silicon oil. **a**, **b** FRET ratio images of slug migrating on top of agar 6 min apart. Note the change in FRET ratio in the slug tip between these two time points. **c** Traces of FRET ratio in tip neck and tail of the slug a shown in **a**. The experiments are representative for seven experiments performed with slugs migrating on agar taken on different days. (**d**, **e**) FRET ratio images of slug expressing the Epac FRET construct under the control of the *pstA* promoter 25 min apart. **f** FRET trace measured in the tip of the slug at the position of the white circle. White scale bar 50 µm. The experiments are representative of nine experiments with *pstA*/Epac1 FRET slugs performed on different days

A major open question is whether cAMP oscillations persist in the slug stage and are responsible for the previously reported optical density waves. Slugs migrating on an agar substrate are difficult to observe in a fluorescence microscope directly since there are massive light reflections from the slug’s surface, the slime sheath. The reflections interfere strongly with the detection of the small FRET signals. Another complication is that slugs migrate away from strong light, which results in them standing up and culminating, making longer term imaging very difficult. For these reasons we have previously developed methods where slugs will migrate in silicon oil that contains high levels of dissolved oxygen that will allow slugs to develop properly. To be able to image them for longer period of time we let them develop in confined conditions^21,42^. We find that cAMP oscillations can be readily observed in migrating slugs under these conditions (Fig. 7). As expected the waves originate in the anterior tip of the slug and propagate rapidly through the back of the slugs (Fig. 7a, b, Supplementary Movie 11). It is difficult to determine precisely where the waves originate, in several cases they seem to be more prominent just behind the tip (Fig. 7c, d, Supplementary Movie 12). Wave propagation speeds are in the order of 200 µm/minute. The frequencies of the waves tend to vary with slug age. In newly formed slugs frequencies tend to be around 4–5 min but in older slugs that have migrated for a few hours the frequencies of the waves maybe slower somewhat less regular and lower in amplitude (Fig. 8a, b, c, Supplementary Movie 13). This is in agreement with our earlier observations on optical density wave propagation in slugs^21^. We were also able to measure FRET oscillations when the Epac1 reporter construct was expressed under the control of the prestalk specific *pstA* promoter^36^. This confirmed that the observed cAMP oscillations are generated by differentiated prestalk cells in the slug tip (Fig. 8d, e, f, Supplementary Movie 14). We observed a number of interesting cases where migrating slugs collide and in these cases the waves appear to interact and sometimes synchronise, which is something we will pursue in forthcoming studies.

**Fig. 9:**
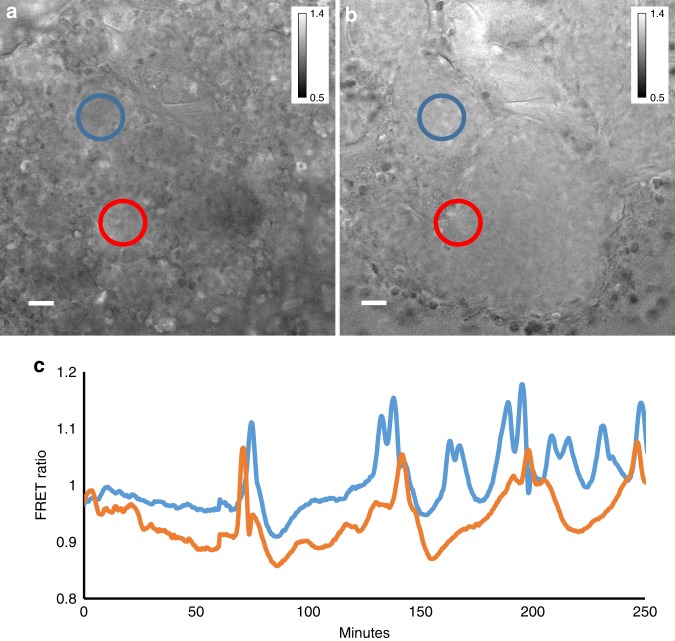
Onset of cAMP oscillations in regenerating slug. **a**, **b** FRET images of regenerating slug taken 200 min apart. Cells of a migrating slug were mechanically mixed to produce a thick cell suspension and regeneration was followed. During regeneration several competing oscillating centres were set up that then fused. **c** FRET changes as function of time measured at two positions indicated by the Red and Blue circles in **a**. White scale bar 50 µm. The experiments are representative of four experiments of slugs regenerating on different days

Finally we wanted to test what happened with cAMP signalling when slugs are forced to regenerate. It is well established that slugs can regenerate when their tissue integrity is destroyed for instance by mechanical stirring of the tissue. In these conditions the prestalk and prespore cells rapidly sort out and reinitiate formation of a new slug. We investigated this situation and found that cAMP waves were readily seen to occur during slug reformation (Fig. 9, Supplementary Movie 15). Waves could be observed, right from the start of the recordings, but there was very complex signalling from centres starting to form and compete in different locations, until a new dominant centre was established again.

**Fig. 10:**
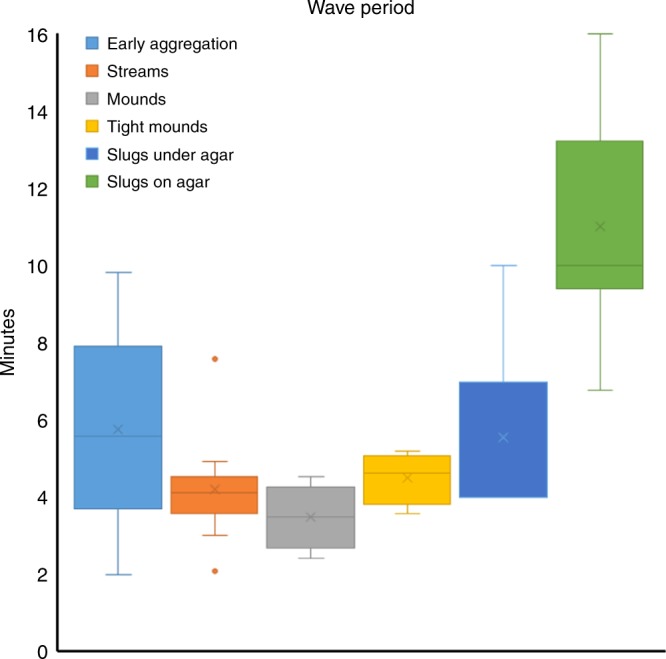
Summary of wave periods at different stages of development. Whisker plot, box showing first and third quartile, the wishers indicate the maximum and minimum values, the line indicates the median and the cross the mean value. The data are from 4–10 experiments for each stage on different days. Early aggregation was after 5–8 h of starvation, streaming aggregates were 8–10 h after starvation, mound stages were 10–12 h after starvation, tight mounds 12–14 h after starvation, slugs were 15–22 h after starvation. Slugs were either confined between a cover slip and agar to allow higher magnification observations in an inverted microscope and to prevent the slugs from moving away from the light to culminate. Alternatively, slugs were allowed to migrate on a thin layer of agar, covered by mineral oil to prevent drying out. The latter only allowed observation at lower magnification and for shorter periods of time since the slugs typically stand up and culminate, which makes measurement of cAMP dynamics difficult

All together the results show the existence of cAMP waves not only during aggregation, but also during the later stages of multicellular development. There are sizable changes in the dynamics of cAMP signalling during development as summarised in Fig. 10 in line with observations made previously about the dynamics of optical density waves in these structures^15,20,21^. The aberrant cAMP signalling dynamics observed in the *tsacaA*, *regA* and *tgrB1*^*−*^/*tgrC1*^−^ mutants showing pronounced developmental defects, strongly support the critical role of proper cAMP wave dynamics in the control and integration of chemotactic cell movements during multicellular *Dictyostelium* morphogenesis.

## Discussion

Previously strong circumstantial evidence had suggested that oscillatory signalling not only controls early aggregation but also late aggregation, the mound and slug stages of development. This evidence was based on the detection of light scattering waves in streaming aggregates, mounds and slugs and the demonstration of periodic cell movements in these structures^21,43^. Further experiments showed these waves could be inhibited by caffeine, a known inhibitor of AcA and that injection of cAMP pulses could initiate and entrain light scattering waves. Inhibition of a temperature sensitive AcA variant resulted in interference or abolishment of these light scattering waves and associated cell movements at the mound and slug stages. The experiments suggested that the oscillatory signals were most likely cAMP oscillations, with AcA being a critical component of the oscillator^20,24^. So far Epac1 based FRET constructs have been used to measure cAMP oscillation in the early aggregation stages of development^18,19^. The measurements reported here now show that cAMP waves can indeed also be detected at the late aggregate, mound and slug stages, as well as during regeneration of slugs. Analysis of the kinetics of the waves has shown that the period of the waves is long during early aggregation, decreases during the late aggregation early mound stages and then lengths again at the slug stages of development (Fig.7). In general it appears that younger slugs show shorter period cAMP oscillations that get longer if slugs migrate for longer periods of time. Migrating slugs show more variable periods of pulses which can range from 4 to 15 min. We also show that during the aggregation and mound stages of development the light scattering density waves appear to be almost in phase with the cAMP oscillations showing that these light scattering waves can serve as a good measure of the dynamics of oscillatory cAMP signalling.

The measurements reported here show that the cAMP oscillations in late aggregates and mounds are inhibited with 5 mM caffeine and in the *tsacaA* mutant at the restrictive temperature, showing that ACA mediates the cAMP oscillations in the later aggregation and mound stages of development. Unfortunately we have not been able to use the *tsAcA* mutant to directly test the role of AcA in the cAMP oscillations in the slug stages, since we have not been able to get migrating slugs that expressed high enough levels of the FRET reporter gene construct to measure cAMP at the permissive and restrictive temperatures. The tsACA most likely produces less cAMP at the permissive temperature than wild-type AcA combined with the fact that expression of *tsacaA* is driven by the actin 15 promoter, whose activity goes down during development. This likely results in a situation where the levels of cAMP produced are just sufficient to aggregate and develop into early mounds, but not sufficient to support slug formation. These observations however strongly reinforce the idea that cAMP produced by AcA plays a key role in slug formation and migration.

We often noted a switch in the apparent pattern of the FRET signals during development. In early aggregation the base level appears low, interspersed with defined spikes of high FRET levels, while during mound formation this pattern changed to high FRET levels with spikes towards lower FRET levels, reflecting saturation of the Epac probe at high cAMP concentrations. These observed changes in cAMP signalling dynamics during development likely reflects quantitative and qualitative changes in the expression of ACA during the later stages of development^44^. Although there are no accurate data on ACA activity in individual cells at different stages of development, gene expression studies have shown that ACA expression becomes restricted to prestalk and anterior like cells^23^. Further dissection of the *acaA* promoter has shown that the *acaA* promotor is regulated by different control elements in prestalk and prespore cell types. Promoter 3 which was shown to be able to rescue development in an *acaA* null strain is active in prestalk cells^45^.

The FRET ratios in slugs are lower in older slugs than in younger slugs and in older slugs the oscillations are slower and less regular than in younger slugs. The reasons for this are so far unresolved, but likely indicates that in the later stages of slug migration the amplitude of the cAMP signals is getting lower.

In previous experimental and theoretical work we have suggested that cAMP waves in the slug are likely to take on the form of scroll waves^5^. That was based on the observation of extensive rotational movement of the cells in the *Dictyostelium* slug, while the cells in the back of the slug migrated forward in a periodic fashion^46^. Our interpretation was that the prestalk region of the slug is essentially like an aggregation centre and the body of the slug like an aggregation stream^47,48^. Our observations of waves in slugs so far have not been able to provide clear evidence for the existence of scroll waves, most of the waves that we have observed appear to be planar. This may be due to the fact that scroll waves do not readily arise in slugs migrating in confined conditions. The latter appears to be supported by the observation that there is relatively little rotational cell movement in the tips of slugs under these conditions. Ideally one would like to take 3D sections of freely migrating slugs and resolve the phase of the oscillations in different parts of the 3D structure. So far we have not been able to perform these measurements and the question whether the waves in tips are scroll waves remains unresolved.

An interesting question is whether slugs can migrate without cAMP oscillations. It has been reported that AK3, a strain that lacks AcA but over expresses the cAMP dependent protein kinase A catalytic subunit can migrate^25^. We have confirmed that this strain can form slugs. It is an open question whether these slugs produce cAMP oscillations, possibly using another mechanisms to achieve oscillations through modulation of cAMP produced by one of the two other adenylcyclases AcB or AcG and or modulation of cAMP degradation and/or transport, or whether there are other guidance mechanisms that can control cell migration in slugs. Unfortunately we have not been able to successfully express the Epac based cAMP reported construct in the AK3 strain and get them to develop. Therefore it remains an open question by which mechanism the cells in these slugs coordinate their movement.

The average FRET ratios in the *regA*^-^ mutant are considerably higher than those found in the Ax2 parent strain at similar stages of development. This indicates that cAMP levels are high in the mutant and that RegA is a major phosphodiesterase involved in the degradation of cAMP in the cells. Oscillations still occur in the small aggregates and mounds formed by the *regA*^−^ mutant, the period is rather short (~2.5 min), but the amplitude of the FRET oscillations is very small. It is to be expected that the high internal cAMP levels result in small amplitude cAMP oscillations, since cAMP cannot be effectively broken down between pulses. It can however currently not be ruled out that the *regA*^−^ mutant produces high amplitude pulses of cAMP that cannot be measured accurately since the FRET probe is close to saturation at the high ambient cAMP levels in this mutant. It is somewhat surprising that there is no significant effect on the period length of the oscillations, something that might have been expected on theoretical grounds, especially if internal cAMP breakdown is a key component of the cAMP oscillator mechanism^49^.

We have previously described very complex wave propagation patterns in streamer-F mutants^50^. There we observed wave trains of two and three waves separated by longer periods of inactivity. At that time we could not distinguish between the possibilities that the measured responses were due to the existence of slow and faster oscillating cells resulting in complex gating behaviours at the population level depending on the strength of coupling of the oscillators, or whether the complex wave patterns were the result of complex oscillator dynamics in individual cells. The measurement of complex cAMP dynamics in single cells of the *tgrB1*^*−*^/*tgrC1*^−^ mutant now shows that at least in this mutant the oscillator can be complex at the individual cell level, showing defined bursting modes. It suggests that TgrB1-TgrC1 cell contact signalling system modulates the coupling strength of cAMP oscillations in individual cells resulting in better synchronisation. Whether changes in cAMP signalling play a major role in the sorting behaviour observed between different Tgr allotypes is an interesting question that remains to be investigated further.

## Methods

### Culture conditions

All strains were grown in standard axenic in HL5 to densities 2–6 × 10^6^ per ml by dilution. To initiate suspension development cells were washed 3 times in KK2 (20 mM KH_2_PO_4_/K_2_HPO_4_, pH 7.0) and suspended to a density of 10^7^ per ml followed by shaking at 180 rpm on an orbital shaker at 21 °C. Alternatively cells were grown in 10 cm petri dishes in 10 ml of HL5 medium. Care was taken to keep cells in the exponential phase of growth (1–8 × 10^6^ per ml).

### cAMP FRET constructs

We have adapted a cAMP specific FRET construct able to measure in vivo cAMP concentrations that is based on the RapGEF, Epac1. The cAMP sensors were based on the H74 turquiose2 EPAC construct^27^. The EPAC-FRET cassette was cut from this vector as a HindIII-XbaI fragment and cloned into the pB17S *Dictyostelium* expression vector^51^. Initial attempts showed that we were unable to express this protein in *Dictyostelium*. We therefore replaced the *turquoise2* sequence with a *Dictyostelium* codon optimised ECFP variant^52^ or with a *Dictyostelium* codon optimised *turquoise2* variant Gene block fragment (Integrated DNA technologies)

TGACTGAAGCTTAAAAATGGTTTCAAAAGGTGAGGAACTTTTTACAGGTGTTGTACCAATCTTGGTAGAGCTTGATGGTGATGTTAATGGACACAAGTTCTCAGTTTCTGGTGAGGGTGAAGGAGATGCTACTTATGGTAAACTTACACTTAAATTCATTTGTACTACTGGTAAATTGCCTGTACCATGGCCAACACTTGTTACTACATTGTCATGGGGAGTTCAATGTTTTGCTCGTTATCCAGATCACATGAAACAACACGATTTCTTCAAGTCAGCTATGCCAGAAGGATATGTTCAAGAGCGTACCATCTTCTTTAAGGATGATGGAAATTACAAAACAAGAGCTGAAGTAAAATTCGAAGGAGATACACTTGTTAATAGAATCGAGTTAAAAGGTATAGATTTCAAAGAAGATGGTAACATATTAGGACACAAATTGGAATACAACTATTTCTCTGATAACGTTTACATAACCGCTGATAAACAAAAGAATGGAATTAAAGCCAATTTCAAGATTCGTCACAATATAGAAGATGGAGGAGTTCAATTAGCTGATCATTACCAACAAAATACCCCAATTGGAGATGGACCTGTATTGCTTCCAGATAATCACTACCTTTCTACCCAAAGTAAATTATCTAAAGATCCTAATGAAAAGCGTGATCACATGGTATTATTAGAGTTTGTAACCGCCGCAAGATATCCATTAGGTATGGATGAGTTGTATAAGTAACTCGAGTGCA

Both fragments were cloned into the original cassette as HindIII-EcoRV fragments.

The H74 construct has been optimised for measurement of FRET via fluorescence lifetime imaging. However after expression of a variant optimised for expression in *Dictyostelium*, we established that ratiometric imaging provided better temporal and spatial resolution. To increase the signal we introduced the Q270E mutation in the human EPAC using site directed mutagenesis^53^. The sequence of the mutation was confirmed by sequencing. To make a cell type-specific expression construct the sensor was cloned in a vector under the control of the *ecmA* promoter by replacing the Gal gene in the *ecmA*-Neo-Gal vector with the Epac cassette. Constructs were transfected into Ax2 cells via electroporation using standard procedures.

For transfection into strains already expressing a neomycin resistance construct like the *tsAcA* strain the cAMP FRET construct was transformed in using co-transfection of blasticidin resistance plasmid and selection in both 10 µg per ml G418 and 5 µg per ml blasticidin.

### Calibration of cAMP FRET sensor

cAMP-FRET calibration curves we obtained from measurements of fluorescence in cell lysates. Calibration in cell lysates worked well. Lysates were prepared by resuspending cells to 5 × 10^7^ per ml in 1 ml of lysis buffer containing 10 mM Tris pH8, 2 mM EDTA, 250 mM Sucrose, passed through a 3 µm isopore TSTP membrane filter twice. The lysates were cleared by centrifugation for or 2 min at 20,000 × g. 400 ul lysate was measured in a 8 well glass bottom microscope chamber using a ×10 objective on a Deltavision microscope, using the microscope settings described above. Increasing amounts of the non hydrolyzable cAMP analogue cAMPS were added and the change in FRET ratio monitored for 5 min. The ratio values were averaged over 4 min. The data from different experiments were normalised to the maximal FRET response at the highest cAMPS concentration and analysed by fitting to a Hill curve with a dedicated Matlab routine.

We attempted to perform calibration in intact cells as well as cell lysates, requiring clamping levels of cAMP and or analogues to known values. Calibration in cells is difficult. Addition of caffeine results in decrease in FRET ratios detected in line with the expectation that levels would drop after addition of a cyclase inhibitor. Addition of the cell permeable EPAC activator 8–CPT–cAMP results in an increase in FRET ratio. However it did not equilibrate to the correct outside concentration, since addition of the secretion inhibitor probenecid resulted in a further increase in FRET signal. For this reason we concluded that absolute calibration in cells would be difficult and report the result throughout the paper as straight FRET ratios.

### Methods to cAMP waves in mounds and slugs

To initiate development for imaging 5 ml of washed cells (10^7^ per ml) were plated on 10 cm bacteriological petri dishes containing 5 ml of 1% agar in KK2. Cells were allowed to settle for 5 min after which the supernatant was drained and the plates incubated at 21 ℃ for the required amount of time. For imaging a small square of agar containing cells was cut out inverted on a glass bottom dish and covered by a thin layer of light silicon oil^54^. For caffeine inhibition experiments the cells were deposited on 25 mm black Millipore filter disks and inverted on a silicone oil filled chamber made by a round plastic spacer. To inhibit the adenylyl cyclase 50 ul of 5 mM caffeine solution in KK2 was carefully pipetted on the top of the Millipore filter.

### Image acquisition and data analysis

Initially images were obtained with a Leica SP8 confocal microscope using a 456 laser line for excitation and emission wascollected between 465–510 nm and 530–580 nm at 1024 × 1024 pixel resolution and saved as 12 bit images. These images were typically quite noisy and required filtering in Image J with a mean filter of radius 1, followed by division to a 32 bit image, removal of outliers with ratio’s larger than 2 followed by 2 × 2 binning.

Later experiments were performed using widefield microscopy on a GE Life Sciences Deltavision microscope using 434/17 nm excitation and emission centred at 465/30 and 525/30 nm. FRET ratios were calculated as the 465/525 ratios. Images were typically collected at 30 s intervals with exposure times of 100 ms at each wavelength with an Olympus 10 × /0.4 or 20 × /0.75 magnification objective. Images were collected with 2 × 2 binning at 12 bit resolution with a CoolSNAP HQ2-ICX285 camera. Images were processed as 16 bit images using Image J and calculation of ratiometric images were performed with 32 bit precision.

### Reporting summary

Further information on experimental design is available in the Nature Research Reporting Summary linked to this article.

## Supplementary information

Description of Additional Supplementary Files

Supplementary Information

Reporting Summary

Supplementary Movie 1

Supplementary Movie 2

Supplementary Movie 3

Supplementary Movie 4

Supplementary Movie 5

Supplementary Movie 6

Supplementary Movie 7

Supplementary Movie 8

Supplementary Movie 9

Supplementary Movie 10

Supplementary Movie 11

Supplementary Movie 12

Supplementary Movie 13

Supplementary Movie 14

Supplementary Movie 15

Supplementary Data 1

## Data Availability

The datasets generated during and/or analysed during the current study are available from the corresponding author on reasonable request. The source data used to produce Figs. 1–10, Supplementary Figures 1 and 2 are provided in the Supplementary Data 1. Representative images from over 300 experiments are available as Supplementary Movies 1–15. The Dictyostelium codon optimised high affinity cAMP FRET construct used in these studies is deposited at the Dictybase stock centre.
