# Supplementary material for: Oscillatory cAMP cell-cell signalling persists during multicellular Dictyostelium development
Source: Commun Biol. 2019 Apr 23;2:139. doi: 10.1038/s42003-019-0371-0 (PMC6478855; doi:10.1038/s42003-019-0371-0)
Supplement: Supplementary file 1 — Description of Additional Supplementary Files [file 42003_2019_371_MOESM1_ESM.pdf]

Description of Additional Supplementary Files

File Name: Supplementary Data 1

Description: This excel file contains the numerical data used to produce figures 1 to 10 and supplementary figures 1 and 2. The data headers describe for each figure the numerical values being used and their units where appropriate.

File Name: Supplementary Movie 1

Description: FRET ratio of aggregating cells, shown in Fig. S2B. Note the Black and white dots indicating fast moving accumulations of the turquoise high affinity Epac probe. Movie speed, 30 frames/sec, time lapse interval 30 seconds, total duration 42 minutes, magnification 60x.

File Name: Supplementary Movie 2

Description: FRET oscillations and optical density in a streaming aggregate, shown in figure 1A. Movie speed, 30 frames/sec, time lapse interval 10 seconds, total duration 120 minutes, magnification 10x.

File Name: Supplementary Movie 3

Description: FRET oscillation of mound before and after addition of 5mM caffeine, shown in figure 1C. Movie speed 30 frames/sec, time lapse interval 15 seconds, total duration 32 minutes, magnification 20x.

File Name: Supplementary Movie 4

Description: FRET oscillations in the ts-acA2 strain at the permissive (21°C) and restrictive temperature (28°C), shown in figure 2A. Movie speed 30 frames/sec, time lapse interval 30 seconds, total duration 225 minutes, magnification 10x.

File Name: Supplementary Movie 5

Description: FRET oscillations in parent Ax2 strain at the permissive (21°C) and restrictive temperature (28°C), shown in figure 2C. Movie speed 30 frames/sec, time lapse interval 30 seconds, total duration 222 minutes, magnification 20x.

File Name: Supplementary Movie 6

Description: Composite of FRET oscillations in *regA*$^-$ mutant, shown in figure 3A and its parent strain Ax2 show in figure 3B. Movie speed 30 frames/sec, time lapse interval 30 seconds, total duration 50 minutes, magnification 10x.

File Name: Supplementary Movie 7

Description: FRET oscillations in end of aggregation stream and position of cells being tracked, in figure 4. Movie speed, 30 frames/sec, time lapse interval 30 seconds, total duration 56 minutes, magnification 10x.

File Name: Supplementary Movie 8

Description: Abrupt changes in FRET signalling during aggregation. Movie speed 30 frames/sec, time lapse interval 30 seconds, total duration 125 minutes magnification 10x.

File Name: Supplementary Movie 9

Description: FRET changes in *tgrB1⁻/tgrC1⁻* mutant showing rapid changes in signalling frequency, shown in figure 5. Movie speed 30 frames/sec, time lapse interval 30 seconds, total duration 90 minutes, magnification 20x.

File Name: Supplementary Movie 10

Description: FRET changes in *tgrB1⁻/tgrC1⁻* mutant aggregation stream and track of three cells showing abrupt changes to fast signalling modes, shown in figure 6. Movie speed 30 frames/sec, time lapse interval 30 seconds, total duration 134 minutes, magnification 10x.

File Name: Supplementary Movie 11

Description: FRET oscillations in a confined slug, shown in figure 7A. Movie speed 30 frames/sec, time lapse interval 30 seconds, total duration 136 minutes, magnification 10x.

File Name: Supplementary Movie 12

Description: FRET oscillations in a confined slug, shown at higher magnification in fig 7C. Movie speed 30 frames/sec, time lapse interval 30 seconds, total duration 166 minutes magnification 20x.

File Name: Supplementary Movie 13

Description: FRET oscillations in slug migration on top of agar shown in figure 8A, B. Movie speed 30 frames/sec, time lapse interval 30 seconds, total duration 110 minutes, magnification 20x.

File Name: Supplementary Movie 14

Description: FRET oscillations in slug expressing the Epac based ECFP cAMP reporter construct under the control of the pstA promoter, shown in figure 8D, E. Movie speed 30 frames/sec, time lapse interval 30 seconds, total duration 118 minutes, magnification 10x.

File Name: Supplementary Movie 15

Description: FRET oscillations in regenerating slug cells, shown in figure 9. Movie speed 30 frames/sec, time lapse interval 30 seconds, total duration 308 minutes, magnification 20x.
