# Supplementary material for: Oscillatory cAMP cell-cell signalling persists during multicellular Dictyostelium development
Source: Commun Biol. 2019 Apr 23;2:139. doi: 10.1038/s42003-019-0371-0 (PMC6478855; doi:10.1038/s42003-019-0371-0)
Supplement: Supplementary file 2 — Supplementary Information [file 42003_2019_371_MOESM2_ESM.pdf]

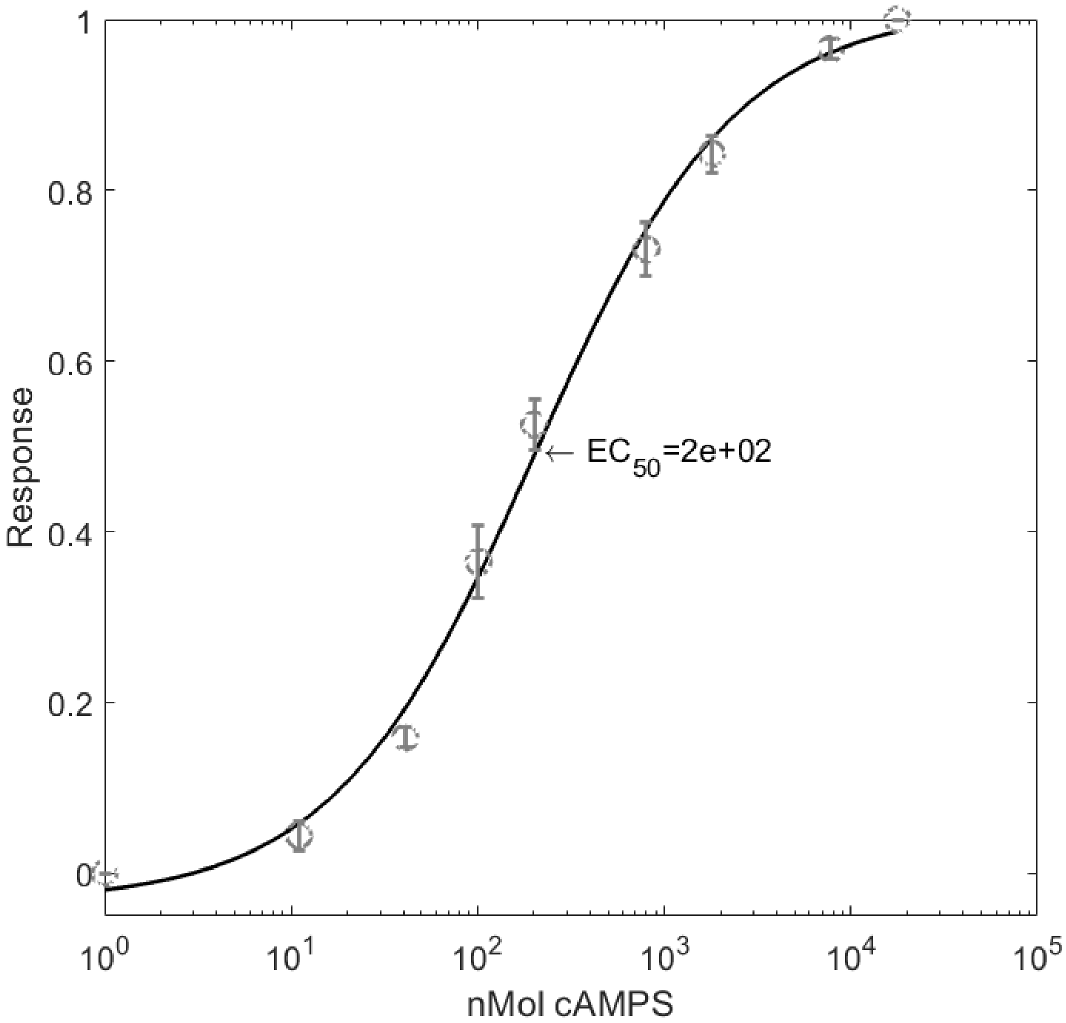

Supplementary Figure 1. Dose response curve of the EPAC turquoise 2 high affinity construct for the cAMP analogue cAMPS. The high affinity Epac construct shows a EC_{50} of 200nM for the nonhydrolyzable cAMP analogue cAMPS. FRET ratios were measured in lysates from aggregation competent cells as described in methods. The curve is constructed from experiments performed on two successive days.

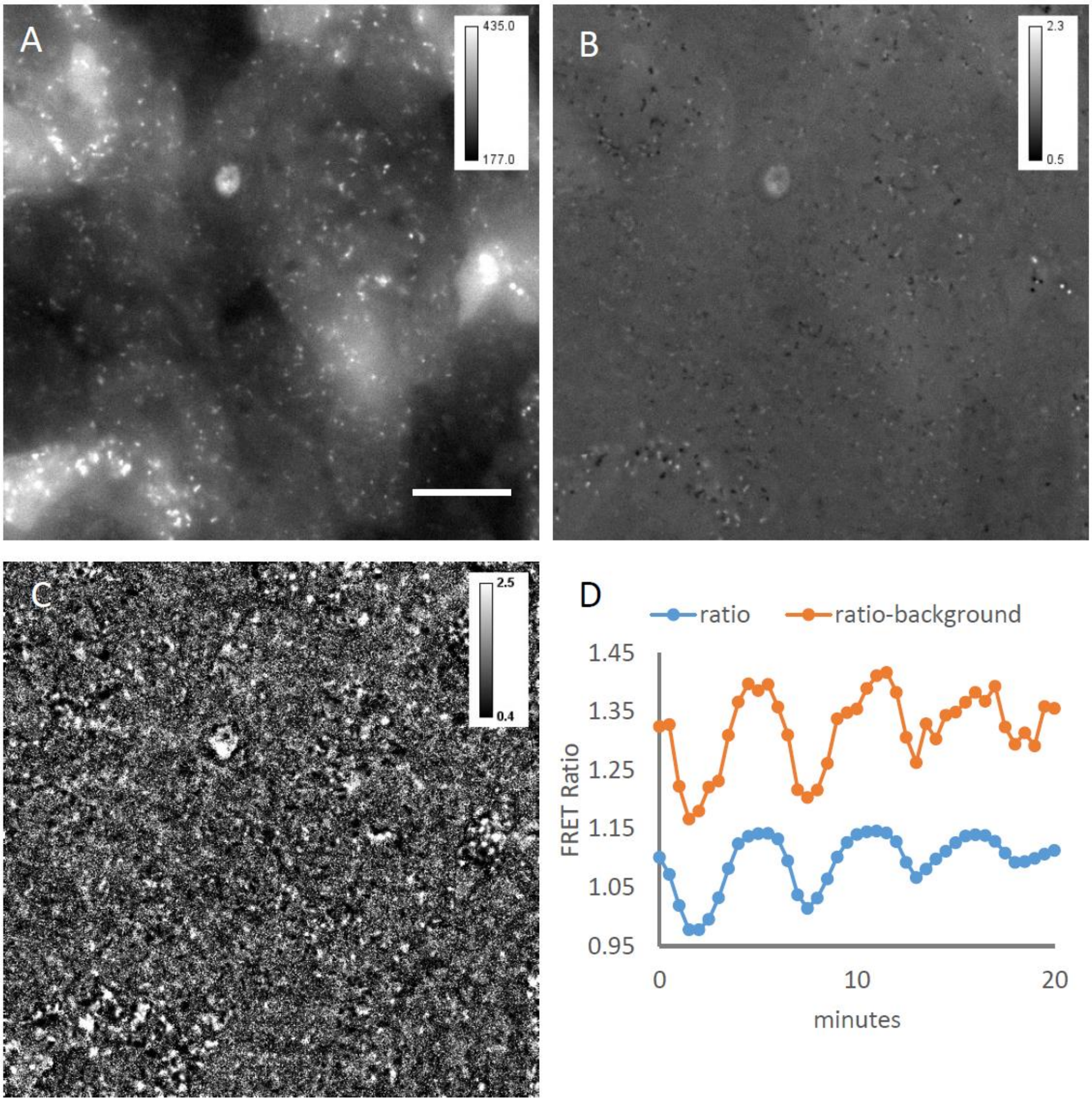

Supplementary Figure 2. Cellular distribution of the high affinity Epac1 construct in aggregating cells. **A**: Image form the blue 465/30 emission channel. **B**: Ratiometric FRET image of the 465/30 and 525/30 channel. **C:** Ratiometric FRET image after subtraction of background fluorescence in the 465/30 and 525/30 before calculation of the ratio. **D:** FRET ratio calculated as average over the whole image of B and C respectively as function of time. Note that both curves show readily detectable oscillations. White scale bar in A, 10 µm.

Supplementary Movie legends

Supplementary Movie 1: FRET ratio of aggregating cells, shown in Fig. S2B. Note the Black and white dots indicating fast moving accumulations of the turquoise high affinity Epac probe. Movie speed, 30 frames/sec, time lapse interval 30 seconds, total duration 42 minutes, magnification 60x.

Supplementary Movie 2: FRET oscillations and optical density in a streaming aggregate, shown in figure 1A. Movie speed, 30 frames/sec, time lapse interval 10 seconds, total duration 120 minutes, magnification 10x.

Supplementary Movie 3: FRET oscillation of mound before and after addition of 5mM caffeine, shown in figure 1C. Movie speed 30 frames/sec, time lapse interval 15 seconds, total duration 32 minutes, magnification 20x.

Supplementary Movie 4: FRET oscillations in the ts-acA2 strain at the permissive (21℃) and restrictive temperature (28°C), shown in figure 2A. Movie speed 30 frames/sec, time lapse interval 30 seconds, total duration 225 minutes, magnification 10x.

Supplementary Movie 5: FRET oscillations in parent Ax2 strain at the permissive (21°C) and restrictive temperature (28°C), shown in figure 2C. Movie speed 30 frames/sec, time lapse interval 30 seconds, total duration 222 minutes, magnification 20x.

Supplementary Movie 6: Composite of FRET oscillations in *regA*$^{-}$ mutant, shown in figure 3A and its parent strain Ax2 show in figure 3B. Movie speed 30 frames/sec, time lapse interval 30 seconds, total duration 50 minutes, magnification 10x.

Supplementary Movie 7: FRET oscillations in end of aggregation stream and position of cells being tracked, in figure 4. Movie speed, 30 frames/sec, time lapse interval 30 seconds, total duration 56 minutes, magnification 10x.

Supplementary Movie 8: Abrupt changes in FRET signalling during aggregation. Movie speed 30 frames/sec, time lapse interval 30 seconds, total duration 125 minutes magnification 10x.

Supplementary Movie 9: FRET changes in *tgrB1*$^-$/*tgrC1*$^-$ mutant showing rapid changes in signalling frequency, shown in figure 5. Movie speed 30 frames/sec, time lapse interval 30 seconds, total duration 90 minutes, magnification 20x.

Supplementary Movie 10: FRET changes in *tgrB1*$^-$/*tgrC1*$^-$ mutant aggregation stream and track of three cells showing abrupt changes to fast signalling modes, shown in figure 6. Movie speed 30 frames/sec, time lapse interval 30 seconds, total duration 134 minutes, magnification 10x.

Supplementary Movie 11: FRET oscillations in a confined slug, shown in figure 7A. Movie speed 30 frames/sec, time lapse interval 30 seconds, total duration 136 minutes, magnification 10x.

Supplementary Movie 12: FRET oscillations in a confined slug, shown at higher magnification in fig 7C. Movie speed 30 frames/sec, time lapse interval 30 seconds, total duration 166 minutes magnification 20x.

Supplementary Movie 13: FRET oscillations in slug migration on top of agar shown in figure 8A, B. Movie speed 30 frames/sec, time lapse interval 30 seconds, total duration 110 minutes, magnification 20x.

Supplementary Movie 14: FRET oscillations in slug expressing the Epac based ECFP cAMP reporter construct under the control of the pstA promoter, shown in figure 8D, E. Movie speed 30 frames/sec, time lapse interval 30 seconds, total duration 118 minutes, magnification 10x.

Supplementary Movie 15: FRET oscillations in regenerating slug cells, shown in figure 9. Movie speed 30 frames/sec, time lapse interval 30 seconds, total duration 308 minutes, magnification 20x.
